# Bioconversion of Terephthalic Acid and Ethylene Glycol Into Bacterial Cellulose by *Komagataeibacter xylinus* DSM 2004 and DSM 46604

**DOI:** 10.3389/fbioe.2022.853322

**Published:** 2022-04-05

**Authors:** Asiyah Esmail, Ana T. Rebocho, Ana C. Marques, Sara Silvestre, Alexandra Gonçalves, Elvira Fortunato, Cristiana A. V. Torres, Maria A. M. Reis, Filomena Freitas

**Affiliations:** ^1^ Associate Laboratory Institute for Health and Bioeconomy, School of Science and Technology, NOVA University Lisbon, Caparica, Portugal; ^2^ UCIBIO—Applied Molecular Biosciences Unit, Department of Chemistry, School of Science and Technology, NOVA University Lisbon, Caparica, Portugal; ^3^ Department of Materials Science, School of Science and Technology, NOVA University Lisbon and CEMOP/UNINOVA, Caparica, Portugal

**Keywords:** bacterial cellulose, bioconversion, PET, terephthalic acid, ethylene glycol

## Abstract

*Komagataeibacter xylinus* strains DSM 2004 and DSM 46604 were evaluated for their ability to grow and produce bacterial cellulose (BC) upon cultivation on terephthalic acid (TA) and ethylene glycol (EG), which are monomers of the petrochemical-derived plastic polyethylene terephthalate (PET). Both strains were able to utilize TA, EG, and their mixtures for BC synthesis, with different performances. *K. xylinus* DSM 2004 achieved higher BC production from TA (0.81 ± 0.01 g/L), EG (0.64 ± 0.02 g/L), and TA + EG mixtures (0.6 ± 0.1 g/L) than strain DSM 46604. The latter was unable to utilize EG as the sole carbon source and reached a BC production of 0.16 ± 0.01 g/L and 0.23 ± 0.1 g/L from TA alone or TA + EG mixtures, respectively. Further supplementing the media with glucose enhanced BC production by both strains. During cultivation on media containing TA and EG, rapid pH drop due to metabolization of EG into acidic compounds led to some precipitation of TA that was impregnated into the BC pellicles. An adaptation of the downstream procedure involving BC dissolution in NaOH was used for the recovery of pure BC. The different medium composition tested, as well as the downstream procedure, impacted the BC pellicles’ physical properties. Although no variation in terms of the chemical structure were observed, differences in crystallinity degree and microstructure of the produced BC were observed. The BC produced by *K. xylinus* DSM 2004 had a higher crystallinity (19–64%) than that of the strain DSM 46604 (17–53%). Moreover, the scanning electron microscopy analysis showed a higher fiber diameter for *K. xylinus* DSM 2004 BC (46–56 nm) than for *K. xylinus* DSM 46604 (37–49 nm). Dissolution of BC in NaOH did not influence the chemical structure; however, it led to BC conversion from type I to type II, as well as a decrease in crystallinity. These results demonstrate that PET monomers, TA and EG, can be upcycled into a value-added product, BC, presenting an approach that will contribute to lessening the environmental burden caused by plastic disposal in the environment.

## Introduction

Polyethylene terephthalate (PET) is a polyester of terephthalic acid (TPA) and ethylene glycol (EG) monomers. PET is the most manufactured thermoplastic globally, due to its remarkable material properties, such as high tensile strength, great chemical resistance, elasticity, electrical insulating properties, and thermostability (Robertson, 2014), which grant its versatility to be used in many industries, such as packaging, textiles, electrical and electronics, and the automotive industry (Webb et al., 2013). However, PET is incredibly resistant to hydrolytic or enzymatic degradation, presenting as a considerable recalcitrant pollutant in the environment and contributing to the alarming worldwide plastic accumulation and pollution problem (Sang et al., 2020). Physical treatments, such as thermal and mechanical procedures, coupled with exposure to chemicals, namely, acids or alkali, are used to depolymerize PET into its monomers (TA and EG) or into low Mw oligomers (Thiounn and Smith, 2020). The resulting TA can be purified and utilized for producing recycled PET, thus valorizing waste PET materials.

The resulting plastic monomers are more easily degraded by microorganisms due to their higher water solubility than the original high molecular weight polymers. Therefore, they can alternatively be used by some bacteria as feedstocks for the production of value-added microbial products, such as polyhydroxyalkanoates (PHAs) (Tiso et al., 2021) and bacterial cellulose (BC) (Zhang et al., 2021). Although this strategy is still underexplored, requiring extensive research for its implementation, it arises as a very promising approach that will simultaneously contribute to mitigate the plastic waste problem, enabling plastic waste to become a resource rather than an environmental burden by adding value to them (Nikolaivits et al., 2021).

BC is a natural polysaccharide synthesized by some species of bacteria, including Gram-negative (e.g., *Acetobacter*, *Gluconacetobacter* (presently *Komagataeibacter*), and *Rhizobium*), as well as Gram-positive bacterial species such as *Sarcina ventriculi* (Carvalho et al., 2019). Among the aforementioned bacteria, *Komagataeibacter* is known to be the most efficient to produce high-quality BC for commercial use (Ruka et al., 2012). Both BC and plant-based cellulose are chemically composed of glucose molecules connected *via* acetal linkages between C1 and C4 carbons (Klemm et al., 2001). Still, there are major differences between them in terms of purity, macromolecular properties, and characteristics. Relative to plant-based cellulose, BC shows higher purity, degree of polymerization, water uptake capacity, crystallinity (up to 96%), biocompatibility, and Young’s modulus (Klemm et al., 2001; Ul-Islam et al., 2012). These distinctive properties favor BC over plant-based cellulose for an array of applications in the food, cosmetics, optoelectronics, and textile industry, as well as in the biomedical field (Fortunato et al., 2016; Carvalho et al., 2019; Wang et al., 2019; Marques et al., 2021).

In accordance with this, this work assessed the capability of *Komagataeibacter xylinus* strains DSM 2004 and DSM 46604 to utilize TA and EG as carbon sources for BC production. Media supplemented with TA and/or EG were tested for the static cultivation of both strains, as well as the same mixtures supplemented with glucose as a co-substrate. The BC pellicles obtained were extracted and characterized in terms of their physical–chemical properties and their nanostructure to evaluate the impact of the medium composition.

## Materials and Methods

### Microorganisms and Media

This study was carried out using two *Komagataeibacter xylinus* strains, namely, DSM 2004 and DSM 46604, purchased from DSMZ (the German Collection of Microorganisms and Cell Cultures). The microorganisms were preserved in glycerol (20%, v/v) (99% Sigma-Aldrich), as a cryoprotectant agent, at -80°C. The inocula were prepared by inoculation of 1 ml of the cryopreserved cultures in the HS (Hestrin–Schramm) medium (Hestrin and Schramm, 1954) (per liter: glucose, 20 g; peptone, 5 g; yeast extract, 5 g; citric acid, 1.15 g; disodium hydrogen phosphate, 2.7 g; pH = 7) and incubation in an orbital shaker, at 30°C and 150 rpm, for 24 h. T-75 flasks (BIOFIL) containing 30 ml medium were inoculated with 20% (v/v) inoculum and incubated statically at 30°C, for 18 days.

The media tested consisted of the non-supplemented HS medium; HS medium supplemented with glucose (20 g/L) (reagent grade, Scharlau), TA (20 g/L) (synthesis grade, Merck), or EG (20 g/L) (Honeywell); and HS medium supplemented with mixtures of glucose, TA, and/or EG. For the preparation of culture media containing TA, sonication in an ultrasonic bath (Bandelin Sonorex Digitec Berlin) of the mixture for 30 min was performed, followed by pH adjustment to 7.0 by addition of 5 M NaOH for complete TA solubilization.

### Analytical Techniques

At the end of the assays, the cellulose membranes were collected from the flasks, and the cultivation broth samples were centrifuged (10,956 × g 15 min, 4°C). The resultant supernatant was collected for glucose, TA, and EG quantification. Broth samples were also collected at the beginning of the assays for nutrient quantification.

The collected cellulose membranes were treated with 0.1 N NaOH, at 80°C, for 20 min (Costa et al., 2017), and neutralized with water in an orbital shaker for 48 h (200 rpm, at 20°C). Wet BC pellicles were weighed after alkaline treatment as well as after lyophilization (ScanVac CoolSafeTM, LaboGene) at -110°C for 48 h, for BC gravimetric quantification.

In some of the assays, there was the formation of a precipitate that got impregnated into the BC pellicles. For BC purification, such pellicles were dissolved in 5 M NaOH at a concentration of 2wt% using the method described by Araújo et al. (2020). Shortly, the BC was dispersed in the alkali solvent system and kept at −20°C for 48 h. During this period, three freeze-thaw cycles were performed in which the thawed suspensions were extensively stirred (at 500 rpm, for 1 h), at 20°C. The resulting solutions were dialyzed with a 12-kDa cut-off membrane (Nadir^®^ dialysis tubing, Carl Roth, Karlsruhe, Germany) against deionized water, at a constant stirring (200 rpm), till neutral pH and constant conductivity values (20 μS/cm) were reached. The dialyzed BC was freeze-dried and gravimetrically quantified.

For glucose and EG quantification, the cell-free supernatant was diluted in sulfuric acid (H_2_SO_4_ 0.01 N) and filtered with modified nylon centrifugal filters (0.2 μm, VWR), at 10,000 rpm for 10 min. Glucose and EG concentration were determined by HPLC with a VARIAN Metacarb column (BioRad) coupled to a refractive index (RI) detector. The analyses were performed at 50°C, with sulfuric acid (H_2_SO_4_ 0.01 N) as the eluent at a flow rate of 0.6 ml/min. Glucose and EG standards were used at concentrations in the range of 0.01—1.0 g/L. For TA quantification, the cell-free supernatant was filtered with modified nylon centrifugal filters (0.2 μm, VWR) and diluted with NaOH 30 mM. TA concentration was determined by HPLC, with an anion exchange column (Ionpac AS11-HC 4.6 × 250 mm equipped with a pre-column) coupled to a conductivity detector. The analyses were performed at 30°C, with NaOH 30 mM as the eluent at a flow rate of 1.5 ml/min. TA standards were used at concentrations in the range of 0.006–1.0 g/L.

### Polymer Characterization

#### Fourier Transform Infrared Spectroscopy

Fourier transform infrared spectroscopy (FTIR) analysis was performed with a Perkin–Elmer Spectrum Two spectrometer. The dried polymer samples were directly analyzed on the FTIR cells. The spectra were recorded between 400 and 4,000 cm^−1^ resolutions with 10 scans, at 20°C**.**

#### Scanning Electron Microscopy

To observe the nanostructure of BC, the lyophilized samples were mounted for observation with scanning electron microscopy (SEM) using double-sided carbon tape and aluminum stubs and sputter-coated with a thin layer of iridium (Q150T ES, Quorum, UK). The analysis was performed in a scanning electron microscope (Hitachi, model *Regulus* 8,220) using an acceleration voltage of 3 kV. The obtained SEM images were processed by ImageJ (NIH image).

#### X-Ray Diffraction

The structural analysis of the samples was performed by X-ray diffraction (XRD) using an X-ray diffractometer (PANalytical X′Pert PRO MRD) with a monochromatic Cu Kα radiation source (45 kV and 40 mA) to scan the samples, which were recorded in a 2θ range from 10° to 90° using a scan rate of 10°/min with a continuous scanning mode. The crystallinity index (CI) was calculated using the XRD deconvolution method as described by Park et al. (2010).

#### Thermogravimetric Analysis

Thermogravimetric (TG) measurements were carried out with a simultaneous thermal analyzer, STA 449 F3 Jupiter from NETZSCH Thermal Analysis (Wittelsbacherstraße, Germany), under nitrogen atmosphere and loading 5 mg of each material into a covered aluminum crucible. The polymers were heated up to 500°C at 10 K/min.

## Results and Discussion

### BC Production

*K. xylinus* strains DSM 2004 and DSM 46604 were screened for their ability to produce BC upon cultivation on the TA and/or EG supplemented HS medium. As shown in Table 1, both strains were able to produce BC in most of the tested medium composition, although with different yields.

**TABLE 1:** Substrate conversion, BC production, and yield of BC on a substrate basis for *K. xylinus* DSM 2004 and DSM 46604 grown on the HS medium supplemented with glucose, TA, and/or EG.

*K. xylinus*	Assay	Substrate consumption (g/L)			BC (g/L)	Yield (g_BC/G_substrate)
		Glc	TA	EG		
DSM 2004	HS	—	—	—	0.29 ± 0.02	—
	HS + Glc	10.40 ± 0.60	—	—	2.10 ± 0.20	0.20 ± 0.03
	HS + TA	—	1.90 ± 0.10	—	0.81 ± 0.01	0.43 ± 0.02
	HS + EG	—	—	4.20 ± 0.80	0.64 ± 0.02	0.15 ± 0.04
	HS + TA + EG	—	(*)	2.90 ± 2.50	0.60 ± 0.10	(*)
		9.30 ± 1.70	1.80 ± 0.60	—	2.00 ± 0.10	0.18 ± 0.05
	HS + Glc + EG	12.60 ± 1.80	—	6.60 ± 1.10	1.00 ± 0.01	0.05 ± 0.01
	HS + Glc + TA + EG	14.40 ± 1.00	(*)	8.50 ± 0.10	1.20 ± 0.03	(*)
DSM 46604	HS	No growth				
	HS + Glc	10.10 ± 0.40	—	—	0.53 ± 0.04	0.05 ± 0.01
	HS + TA	—	0.90 ± 0.30	—	0.16 ± 0.01	0.18 ± 0.08
	HS + EG	No growth				
	HS + TA + EG	—	(*)	0.45 ± 0.50	0.23 ± 0.10	(*)
	HS + Glc + TA	9.80 ± 1.20	1.60 ± 0.05	—	0.70 ± 0.20	0.06 ± 0.02
	HS + Glc + EG	9.30 ± 1.40	—	6.10 ± 0.50	0.40 ± 0.02	0.03 ± 0.02
	HS + Glc + TA + EG	15.40 ± 1.00	(*)	8.20 ± 0.20	0.76 ± 0.10	(*)

(*)- not quantified due to TA precipitation.

*K. xylinus* DSM 2004 grew on all tested media. As expected, the highest BC production was observed in the glucose-supplemented medium (2.1 ± 0.2 g/L), which is in the range of the values reported for *K. xylinus* strains in the HS medium with glucose (0.24–3.1 g/L) (Mikkelsen et al., 2009; Yang et al., 2016; Ogrizek et al., 2021). The culture was able to grow on both TA- and EG-supplemented media, producing BC at concentrations of 0.81 ± 0.01 g/L and 0.64 ± 0.02 g/L, respectively. Considering that only residual growth was observed on the non-supplemented HS medium (0.29 ± 0.02 g/L), probably by the utilization of peptone, yeast extract and/or citric acid present on the HS medium served as substrates. These results show that strain DSM 2004 was able to utilize TA and EG as carbon sources for BC production. This was confirmed by the consumption of 1.9 ± 0.1 g/L of TA and 4.2 ± 0.8 g/L of EG during the assays (Table 1). The highest yield on a substrate basis was obtained for TA (0.43 ± 0.02 g BC/g substrate), which was considerably higher than the values obtained for glucose or EG (0.20 ± 0.03 and 0.15 ± 0.04 g BC/g substrate, respectively).

**FIGURE 1:**
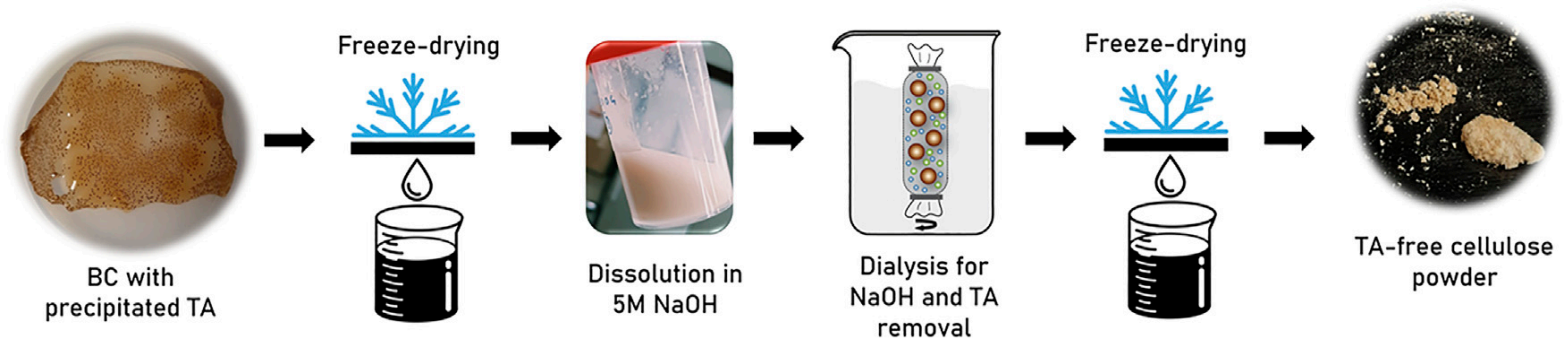
Schematic representation of the strategy for the elimination of precipitated TA from BC pellicles produced in the HS medium supplemented with EG and TA mixtures.

On the other hand, upon cultivation on the HS medium supplemented with TA and EG mixtures, strain DSM 2004 had a BC production of 0.60 ± 0.10 g/L, which is similar to that on EG (0.64 ± 0.02 g/L). However, in that assay, it was not possible to quantify TA due to its precipitation during cultivation that was probably caused by the decrease in the medium’s pH due to the metabolization of EG into acidic metabolites produced, namely, glycolic and oxalic acids (Viinamöki et al., 2015). Since TA is only soluble in neutral to basic solutions (Palme et al., 2017), insoluble particles formed and became impregnated into the synthesized BC pellicles. TA precipitation during the TA + EG supplemented assays interfered with the gravimetrical quantification of the produced BC. Hence, to overcome this, a strategy was implemented to eliminate TA precipitate from the BC pellicle. A schematic of the approach is represented in Figure 1 The produced BC pellicles were dissolved in 5 M NaOH using the freeze-thaw procedure described by Araújo et al. (2020) (Araújo et al., 2020), resulting in a solution of BC and TA that was dialyzed with a 12-kDa cutoff membrane against deionized water. The dialysis permitted the low molecular weight TA molecule to be eliminated from the solution, while the TA-free BC was retained inside the membrane due to its higher molecular weight and quantified by freeze-drying.

BC production on the HS medium supplemented with glucose and TA (2.0 ± 0.1 g/L) was identical to that obtained in the glucose-supplemented medium (2.1 ± 0.2 g/L) (Table 1), which suggests no gain on utilizing TA together with glucose. On the other hand, supplementing the medium with EG (in the glucose + EG and glucose + TA + EG assays) improved BC production (1.00 ± 0.01 g/L and 1.20 ± 0.03 g/L, respectively) compared to EG alone or TA + EG (0.64 ± 0.02 and 0.60 ± 0.1, respectively), but it was significantly lower than production from glucose alone. In those assays, despite the complete glucose consumption, the lower BC production resulted in reduced yields on the substrate (Table 1). These results suggest that the presence of EG in the culture medium induced metabolic pathways other than cell growth or BC synthesis. Nevertheless, the obtained BC production in the assays containing TA + EG was only slightly lower than the values reported for BC production by the bacterial isolate *Taonella mepensis* WT-6 from terylene ammonia hydrolysate (1.75–2.42 g/L) containing TA and EG (Zhang et al., 2021).

Concerning *K. xylinus* DSM 46604, it presented no cell growth on the non-supplemented HS medium or on the EG-supplemented medium (Table 1). Moreover, it presented significantly lower BC production in all other tested media than strain DSM 2004. However, it was able to produce BC when grown on TA (0.16 ± 0.01 g/L), despite the lower production than that observed in the glucose-supplemented medium (0.53 ± 0.04 g/L) (Table 1). Combining glucose and TA as substrates resulted in a higher BC production (0.70 ± 0.20 g/L), which demonstrates that strain DSM 46604 was able to utilize both carbon sources simultaneously. Contrary to strain DSM 2004, the presence of EG in the cultivation medium was not detrimental for cell growth and BC synthesis, with slightly higher or similar production being attained in the TA + EG and glucose + TA + EG assays (0.23 ± 0.1 and 0.76 ± 0.1 g/L, respectively), compared to the TA alone and glucose + TA assays. Moreover, analogous to DSM 2004, precipitation of TA was observed for assays containing TA and EG due to the acidification of the medium. Therefore, purification of the BC pellicles was performed, as previously described, to eliminate the precipitated TA, and obtain pure BC for quantification and further characterization.

Although *K. xylinus* DSM 2004 and 46604 were able to synthesize BC using TA and/or EG, the production is lower than that observed for other *K. xylinus* strains on multiple substrates, such as waste mango pulp (6.32 g/L) (García-Sánchez et al., 2020), grape bagasse (8 g/L) (Vazquez et al., 2013), glycerol from biodiesel production (10 g/L) (Vazquez et al., 2013), cotton cloth hydrolysate (10.8 g/L) (Hong et al., 2012), and wheat straw acid hydrolysate (15.4 g/L) (Hong et al., 2011). Most of such feedstocks are sugar-rich materials that are easily assimilable by the cells, in contrast to TA or EG, which are not so easily metabolized by the cells. Nevertheless, these results demonstrate the synthesis of BC from PET monomers TA and EG, although the process requires further efforts for its optimization, namely, regarding the optimization of the medium composition (e.g., defining optimal carbon and nitrogen sources concentrations) and the cultivation conditions, such as the pH, temperature, and oxygen supply.

### BC Characterization

The BC pellicles obtained in all assays were characterized for their morphology and physical–chemical properties.

#### Morphology

**FIGURE 2:**
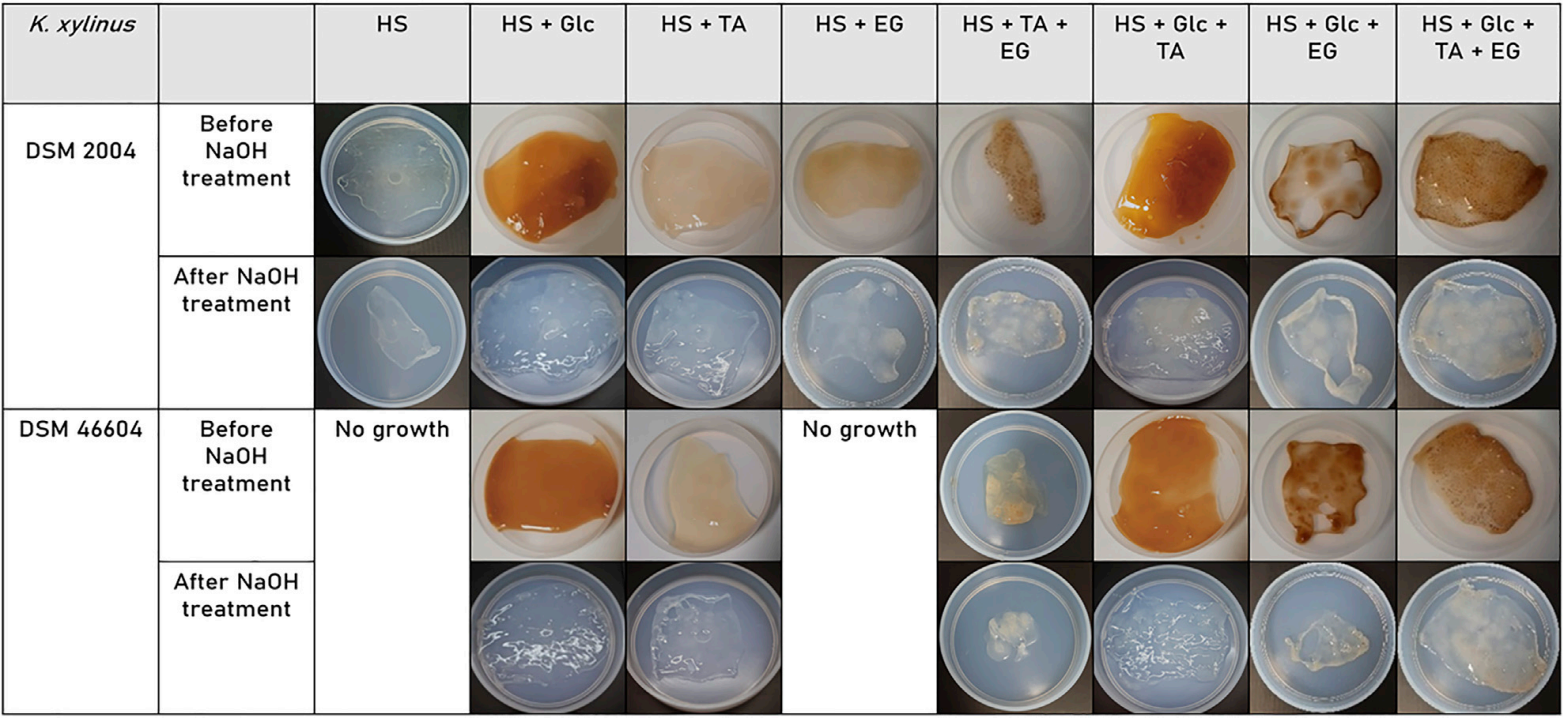
BC pellicles produced by the cultivation of *K. xylinus* strains DSM 2004 and DSM 46604 on the HS medium supplemented with glucose, TA, and/or EG.

Figure 2 shows the wet BC pellicles in each cultivation assay, as produced and after NaOH treatment. The produced BC pellicles displayed an orange/yellowish tint before the alkaline treatment and became translucent afterward. This demonstrates the treatment was efficient on the removal of bacterial cell debris and medium remnants from the pellicles. The size and thickness of the pellicles increased accordingly to the bacterial growth and BC production (Zhong, 2020) described previously. After freeze-drying, the membranes presented a papery texture and lost their transparency, becoming white and opaque, as has been previously reported in the literature (Vasconcellos and Farinas, 2018).

**FIGURE 3:**
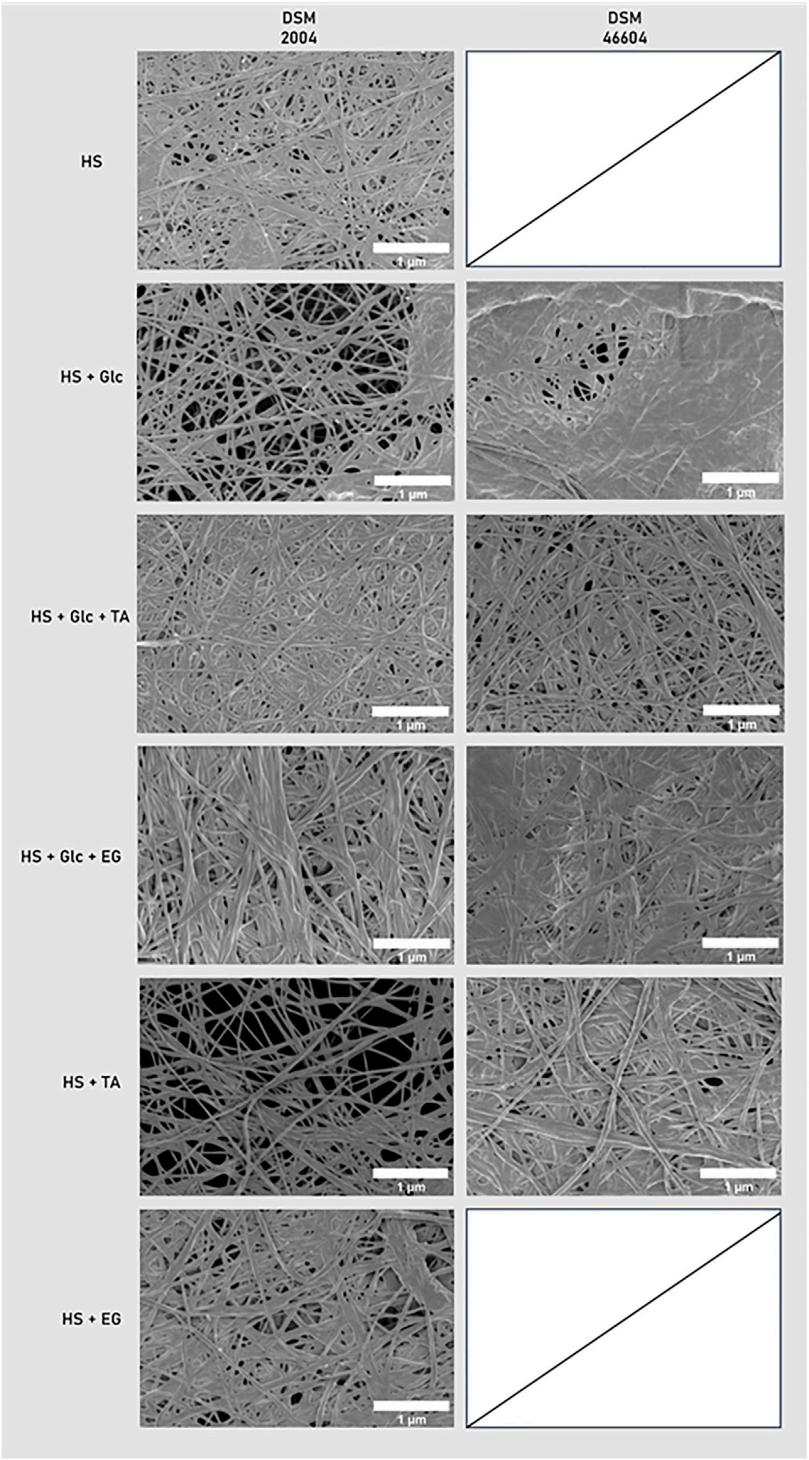
SEM images of BC grown in glucose, TA, and EG by *K. xylinus* DSM 2004 and DSM 46604.

The SEM images of the freeze-dried BC pellicle surface are shown in Figure 3 for both *K. xylinus* strains. The BC obtained after elimination of the TA precipitate was not characterized since the native 3D network was disrupted by the process.

The nanostructure displayed by all samples is similar to that described for dried BC membranes, namely, a three-dimensional porous network of continuous nanofibers (Swingler et al., 2021; Fortunato et al., 2016). The fibers’ diameter ranged between 23 and 90 nm (Table 2), which is within the values reported for BC (20–100 nm) produced by different bacteria (Szymańska-Chargot et al., 2011; Gayathri and Srinikethan, 2019; Wang et al., 2019). Nevertheless, some differences are noticed between the samples, which might be correlated with the producing strain, as well as with the carbon source utilized for cultivation. On average, the BC produced by *K. xylinus* DSM 2004 presented a slightly higher fiber diameter (27–90 nm) than that of strain DSM 46604 (23–68 nm). Moreover, the latter structures are shown to be more tightly packed for all media tested, showing a higher number of fused fibers (Figure 3).

**TABLE 2:** Fiber diameter, crystallinity index (CI), weight loss, char yield, and degradation temperature (T_deg_) of BC grown in glucose, TA, and EG by *K. xylinus* DSM 2004 and DSM 46604.

Strain	Assay	Fiber diameter (nm)	CI (%)	Weight loss (%)			Char yield (%)	T_deg_ (°C)
				30–100°C	225–375°C	380–500°C		
2004	HS	27–90	64	3	67	7	23	337
	HS + Glc	29–75	63	6	56	12	27	327
	HS + Glc + TA	34–84	45	5	59	9	27	334
	HS + Glc + EG	35–70	61	6	58	12	25	331
	HS + Glc + TA + EG	(*)	19	7	52	14	27	325
	HS + TA	33–68	57	4	56	13	27	333
	HS + EG	41–74	40	5	60	8	27	323
46604	HS + Glc	23–68	33	6	56	13	25	328
	HS + Glc + TA	28–55	53	5	55	13	27	331
	HS + Glc + EG	29–66	37	6	62	8	23	338
	HS + Glc + TA + EG	(*)	17	4	61	5	29	333
	HS + TA	33–63	48	4	48	10	37	315

(*)- not quantified due to TA precipitation.

#### Chemical Structure

**FIGURE 4:**
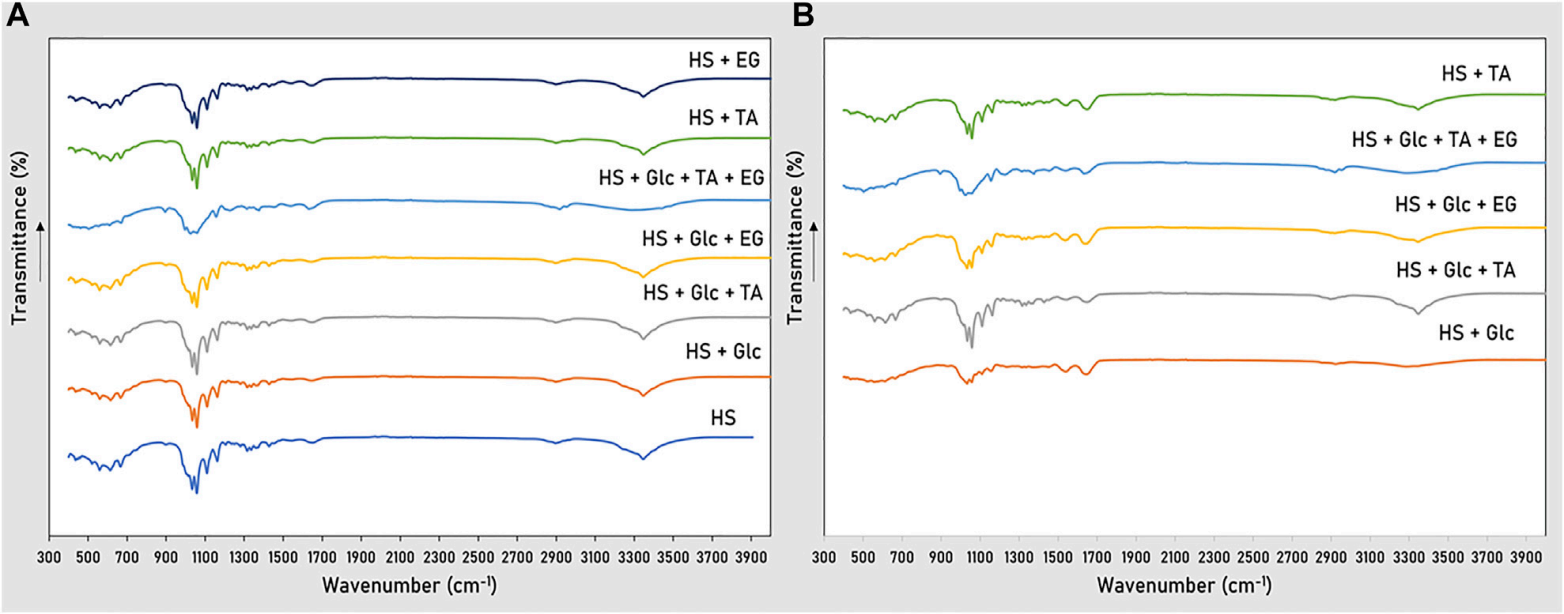
FTIR spectra of the chemical groups present in BC grown in glucose, TA, and EG by *K. xylinus* DSM 2004 **(A)** and DSM 46604 **(B)**.

Similar FTIR spectra were obtained for the BC produced from glucose, TA and/or EG by *K. xylinus* DSM 2004 (Figure 4A) and DSM 46604 (Figure 4B), suggesting that the use of TA and/or EG had no significant influence on the chemical structure of the biopolymer synthesized. All spectra show the characteristic bonds reported for BC (Gea et al., 2011). The intense peak at 3,346 cm^−1^ can be attributed to the stretching of hydroxyl groups in cellulose (Deng et al., 2003). The presence of asymmetric stretching for CH_2_ was revealed by the band at 2,896 cm^−1^ (Oh et al., 2005). Also, the bending of HCH and OCH was observed at 1,427 cm^−1^ (Oh et al., 2005). In addition, the spectra showed peaks at 1,360–1,315 cm^−1^ that may be linked to C–H bonds (Gea et al., 2011). The stretching band detected at 1,162 cm^−1^ is related to C–O–C asymmetric stretching and CH deformation (Kačuráková et al., 2002). Moreover, the peak appearing at 1,109 cm^−1^ can be associated with the stretching of C–C rings in polysaccharides (Movasaghi et al., 2008). However, some differences can be seen in the spectra of the BC samples produced in the Glc + TA + EG supplemented media, namely, a lower intensity of the peaks appearing at the regions around 960–1,100 cm^−1^ and 3,346 cm^−1^, which is probably due to the processing that the pellicles were subjected for the elimination of the precipitated TA. Furthermore, no peaks associated with the FTIR spectrum of TA (Supplementary Appendix SA) (Téllez et al., 2001) were noticed on the spectra of the BC samples, thus confirming the purification procedure was efficient.

#### Crystallinity

**FIGURE 5:**
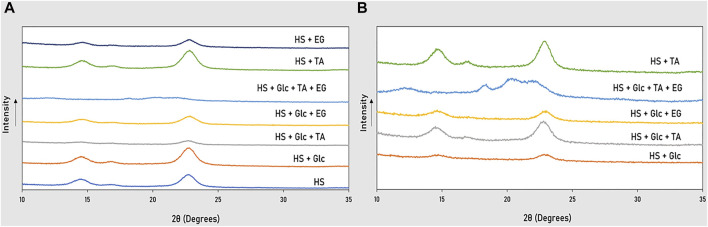
XRD diffractograms of BC grown in glucose, TA, and EG by *K. xylinus* DSM 2004 **(A)** and DSM 46604 **(B)**.

The structural analysis performed by XRD (Figure 5) allowed the determination of the crystallinity index (CI) of the BC produced by each strain from the different substrates (Table 2). Most samples exhibited some or all of the main reflections of the X-ray diffraction pattern of crystalline BC, presenting typical peaks for the crystalline phase with different intensities, specifically three narrow humps located at 2θ = 15, 17, and 23°, corresponding to the (1–10), (110), and (200) crystal planes (Ye et al., 2019). However, the BC produced by cultivation on Glc + TA + EG by both bacterial strains that were subjected to precipitate removal showed a different profile, exhibiting a pattern of diffraction that is the characteristic of the polymorphic transformation of cellulose I to cellulose II (Pandey et al., 2014), specifically a broad hump within the 2θ = 20–22° region, as well as some degree of crystallinity, confirmed by two shifted small peaks in the crystalline zone near 12 and 18°. The difference in reflections by these BC samples can be explained by the fact that they were subjected to dissolution in NaOH, which has been reported to decrease BC crystallinity by cleaving inter- and intramolecular bonds and destroying the crystalline region from penetrating the amorphous area of the polymer (Pandey et al., 2014).

The CI values are well in accordance with the XRD patterns of the different BC samples (Table 2). The results differed depending on the bacterial strain used, as well as the medium components, since these conditions have been described to impact BC properties, along with pH and oxygen delivery (Pourramezan et al., 2009). BC is formed by a multistep process of production and crystallization where the sub-fibrils of cellulose are extruded linearly through the pores at the surface of the cell membrane of the bacteria, where they are crystallized into microfibrils (Wang et al., 2019). Moreover, the rate at which the bacteria synthesizes BC also impacts the biopolymer’s crystallinity (Ruka et al., 2012), which might be the case for our investigation, where *K. xylinus* DSM 2004 presented, in average, the production of BC of higher CI than *K. xylinus* DSM 46604 (19–64% against 17–53%, respectively). Moreover, the different cultivation media apparently impacted the CI of the BC that is produced differently for each bacterial strain. For strain DSM 2004, the highest CI values were found for the BC synthesized in the non-supplemented HS medium and the glucose-supplemented media (64 and 63%, respectively), decreasing for the BC produced in the presence of TA (45–57%) or EG (40–61%) (Table 2). An opposite trend was noted for strain 46604, for which the lowest CI value was observed for the BC produced in the glucose medium (33%), increasing for the biopolymers produced in the TA (48–53%) or EG (37%) supplemented media (Table 2), which may indicate that TA allowed a higher BC crystallization of that strain. These results indicate that CI is influenced by medium composition, as reported in previous studies that proved different additives (e.g., agar, carboxymethylcellulose (CMC), and sodium alginate) in the culture medium impact the productivity, crystallinity, and crystal size of BC (Cheng et al., 2009).

#### Thermal Properties

**FIGURE 6:**
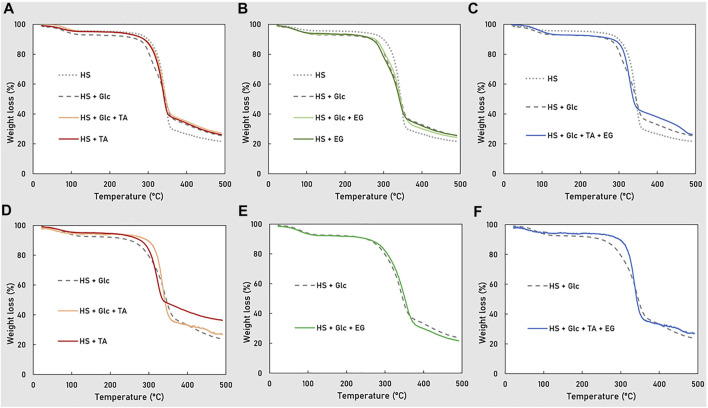
TGA thermograms of BC produced by *K. xylinus* DSM 2004 **(A–C)** and DSM 46604 **(D–F)**, in the different tested media.

The thermal decomposition behavior, namely, the thermograms and the degradation temperature, of the BC produced by each strain upon cultivation on glucose, TA, and/or EG is presented in Figure 6 and Table 2. All samples present a similar profile, experiencing three weight loss events (Figure 6), in accordance with the reported studies for BC (Gayathri and Srinikethan, 2019; Zhang et al., 2021). The first degradation step (3–7% weight loss), which occurred between 30 and 100°C, can be attributed to the loss of crystal water existing in BC samples (Zhang et al., 2021). The second and most significant weight loss (48–67%) was observed between 225 and 375°C, and is related to the pyrolysis of BC, where the main degradation of the polymer occurs (George et al., 2005). The last weight-loss step (5–14%), occurring between 380 and 500°C, corresponds to the degradation of reminiscent microorganisms or protein present in the samples (Zhang et al., 2021). The samples char yield at 490°C ranged between 23 and 37 (Table 2).

For strain DSM 2004, the BC produced in the non-supplemented medium (Figure 6A) had the highest T_deg_ value (337°C) and the lowest char yield (23%) (Table 2). In contrast, cultivation on the glucose-supplemented medium resulted in the BC with higher char yield (27%) and lower T_deg_ (327°C). The profiles for the EG and the Glc + EG samples (Figure 6B) were similar to that of the glucose-supplemented medium (Figure 6A), while the BC produced upon cultivation on TA or Glc + TA (Figure 6A) shows Tdeg values (333–334°C) similar to those of the BC sample from the HS medium, but the char yield (27%) was identical to that of the HS + Glc assay. The profile for BC grown in the Glc + TA + EG mixture (Figure 6C) confirmed that the thermal properties of BC were not significantly impacted by TA precipitate removal in comparison to BC grown in Glc. A similar trend was noticed for strain DSM 46604, for which the TGA profiles for the BC samples produced in the presence of glucose (Figure 6D) were similar to those of the EG assay (Figure 6E), while the samples obtained in the TA and Glc + TA assays (Figure 6D), as well as Glc + TA + EG (Figure 6F), differed. Nevertheless, the T_deg_ values found for all assays were in the 315–338°C range (Table 2), which is among the values reported for BC (312–356°C) produced by different bacteria (George et al., 2005; Jia et al., 2017; Bekatorou et al., 2019). Despite identified differences, these results show there was no significant impact on the samples’ thermal degradation profile upon cultivation on TA and/or EG.

## Conclusion

*Komagataeibacter xylinus* strains DSM 2004 and DSM 46604 demonstrated their ability to grow on media supplemented with TA and/or EG, the monomers of PET. *K. xylinus* DSM 2004 displayed the most promising performance, being able to utilize both TA and EG as sole carbon sources. Although the BC produced in all tested media had identical chemical structure and thermal behavior, their crystallinity and nanostructure varied with the producing bacterial strain, as well as with the medium composition. These promising findings pave the way for the upcycling of PET degradation monomers into a high-value biopolymer, thus contributing to the reduction of the plastics’ harmful impact to the environment.

## Data Availability

The raw data supporting the conclusions of this article will be made available by the authors, without undue reservation.
