# Supplementary material for: Bioconversion of Terephthalic Acid and Ethylene Glycol Into Bacterial Cellulose by Komagataeibacter xylinus DSM 2004 and DSM 46604
Source: Front Bioeng Biotechnol. 2022 Apr 5;10:853322. doi: 10.3389/fbioe.2022.853322 (PMC9036990; doi:10.3389/fbioe.2022.853322)
Supplement: Supplementary file 1 [file Image1.pdf]

Appendix A

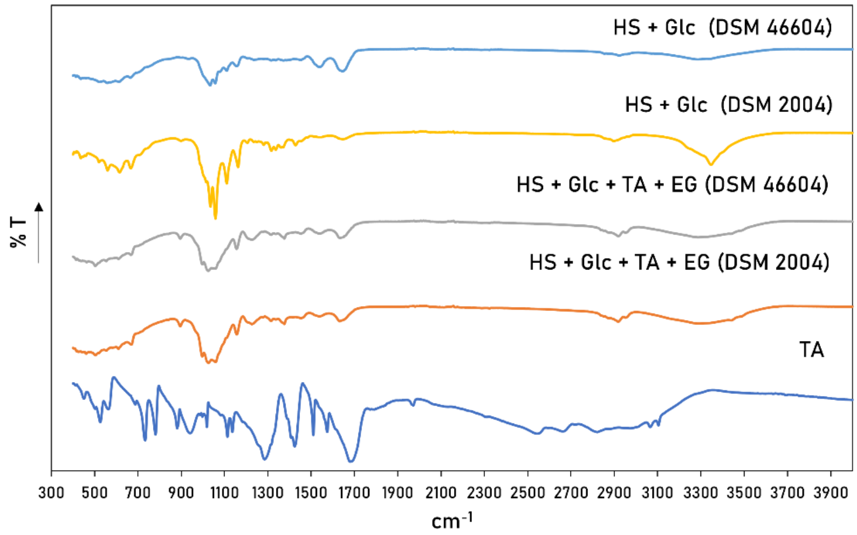

Appendix A- FTIR spectra of the chemical groups present in commercial TA and in BC grown in glucose, TA and EG by *K. xylinus* DSM 2004 and DSM 46604.
